# The Construction and Validation of a Novel Ferroptosis-Related Gene Signature in Parkinson’s Disease

**DOI:** 10.3390/ijms242417203

**Published:** 2023-12-06

**Authors:** Tingting Liu, Haojie Wu, Jianshe Wei

**Affiliations:** Institute for Brain Sciences Research, School of Life Sciences, Henan University, Kaifeng 475004, China; ltt0808@henu.edu.cn (T.L.);

**Keywords:** Parkinson’s disease, ferroptosis, mitochondria, substantia nigra

## Abstract

As a newly discovered regulated cell death mode, ferroptosis is associated with the development of Parkinson’s disease (PD) and has attracted much attention. Nonetheless, the relationship between ferroptosis and PD pathogenesis remains unclear. The GSE8397 dataset includes GPL96 and GPL97 platforms. The differential genes were analyzed by immune infiltration and Gene Set Enrichment Analysis (GSEA) (*p* < 0.05), and differential multiple |logFC| > 1 and weighted gene coexpression network analysis (WGCNA) were used to screen differential expression genes (DEGs). The intersection with 368 ferroptosis-related genes (FRGs) was conducted for gene ontology/Kyoto encyclopedia of gene and genome (GO/KEGG) enrichment analysis, gene expression analysis, correlation analysis, single-cell sequencing analysis, and prognosis analysis (area under the curve, AUC) and to predict relevant miRNAs and construct network diagrams using Cytoscape. The intersection genes of differentially expressed ferroptosis-related genes (DEFRGs) and mitochondrial dysfunction genes were validated in the substantia nigra of MPTP-induced PD mice models by Western blotting and immunohistochemistry, and the protein-binding pocket was predicted using the DoGSiteScorer database. According to the results, the estimated scores were positively correlated with the stromal scores or immune scores in the GPL96 and GPL97 platforms. In the GPL96 platform, the GSEA showed that differential genes were mainly involved in the GnRH signaling pathway, B cell receptor signaling pathway, inositol phosphate metabolism, etc. In the GPL97 platform, the GSEA showed that differential genes were mainly involved in the ubiquitin-mediated proteolysis, axon guidance, Wnt signaling pathway, MAPK signaling pathway, etc. We obtained 26 DEFRGs, including 12 up-regulated genes and 14 down-regulated genes, with good correlation. The area under the prognostic analysis curve (AUC > 0.700) showed a good prognostic ability. We found that they were enriched in different neuronal cells, oligodendrocytes, astrocytes, oligodendrocyte precursor cells, and microglial cells, and their expression scores were positively correlated, and selected genes with an AUC curve ≥0.9 were used to predict miRNA, including miR-214/761/3619-5p, miR-203, miR-204/204b/211, miR-128/128ab, miR-199ab-5p, etc. For the differentially expressed ferroptosis–mitochondrial dysfunction-related genes (DEF-MDRGs) (*AR*, *ISCU*, *SNCA*, and *PDK4*), in the substantia nigra of mice, compared with the Saline group, the expression of AR and ISCU was decreased (*p* < 0.05), and the expression of α-Syn and PDK4 was increased (*p* < 0.05) in the MPTP group. Therapeutic drugs that target SNCA include ABBV-0805, Prasinezumab, Cinpanemab, and Gardenin A. The results of this study suggest that cellular DEF-MDRGs might play an important role in PD. *AR*, *ISCU*, *SNCA*, and *PDK4* have the potential to be specific biomarkers for the early diagnosis of PD.

## 1. Introduction

Parkinson’s disease (PD) is one of the most common neurodegenerative diseases. The main clinical manifestations include motor symptoms (bradykinesia, rigidity, resting tremor, postural instability) and nonmotor symptoms (hyposmia, depression, sleep disorders) [1]. The most prominent pathological features of PD are the loss of dopaminergic (DAergic) neurons and the abnormal accumulation of α-synuclein (α-Syn) in the substantia nigra (SN). It has been found that the main motor symptoms of PD are due to dopamine deficiency and the degeneration of the nigrostriatal pathway that releases dopamine, leading to an imbalance of excitatory (acetylcholine) and inhibitory (dopamine, DA) neurotransmitters in this region [2]. Recent studies have shown that high levels of iron play a key role in the onset and progression of neurodegenerative diseases by activating oxidative stress and other processes. In addition, the dysfunction of the iron metabolism has been found to play a key role in the onset and progression of neurodegenerative diseases [3]. In PD patients, iron–melanin interaction promotes DA neuron degeneration, and the iron content in SN was significantly higher compared with normal controls in previous studies [4,5].

In recent years, studies have found that there is excessive iron deposition in the substantia nigra pars compacta (SNpc) of PD [6]. The abnormal accumulation of iron can generate hydroxyl radicals through a Fenton reaction and induce neuronal damage, which may partly explain the death of nerve cells. However, iron accumulation also points to a novel form of iron-dependent programmed cell death called ferroptosis. Ferroptosis is a new type of iron-dependent regulatory cell death, which is different from apoptosis, autophagy, and necrosis [7]. It is characterized by iron-dependent accumulation of lethal lipid reactive oxygen species (ROS). The essence of ferroptosis is that glutathione depletes, glutathione peroxidase (GPX4) activity decreases, lipid oxides are not metabolized by the GPX4-catalyzed glutathione reductase reaction, and then Fe^2+^ oxidize lipids to produce ROS, thus promoting the occurrence of ferroptosis [8]. The role of heavy metals, such as copper, in promoting the onset of PD is supported by several epidemiological studies. In addition to iron and zinc, copper is the most abundant metal ion in the central nervous system (CNS), and the highest copper content in the brain is in the locus coeruleus and SN. Copper is a reduction–oxidation (REDOX) active metal that acts as a cofactor and/or structural component of a variety of enzymes that are involved in cell respiration, free radical detoxification, iron metabolism, and the synthesis of neurotransmitters, neuropeptides, and hormones. It is also related to angiogenesis, neuroregulation, and other processes. The homeostasis of copper in the brain is regulated by neurons, astrocytes, and the blood–brain barrier (BBB) [9]. In addition to being a cofactor in many REDOX enzymes and participating in melanin formation, copper has been shown to play a key role in synapses through its interactions with synaptic proteins and neurotransmitter receptors [10]. In summary, by modulating synaptic activity, excitotoxic cell death, and signaling cascades induced by neurotrophic factors, copper participates in several neural cell functions. In PD, one widely accepted mechanism by which copper is involved in the pathogenesis of PD is through its ability to increase oxidative stress by catalyzing toxic REDOX reactions involving oxygen derivatives, and, furthermore, unstable copper ions in the brain are a major source of DA oxidation [11]. At the same time, the physiological and pathological significance of the interaction between α-Syn and copper has been widely reported [12,13]. The neurotoxicity of manganese (Mn) may stem from its affinity for high levels of neuromelanin regions, as well as its tolerance to multiple oxidative environments, leading to the self-oxidation of DA and the production of ROS. Mn often causes damage to DAergic neurons through the oxidative stress response, mitochondrial function, protein homeostasis antagonism, destruction of DAergic synaptic function, activation of endoplasmic reticulum stress, activation of apoptosis, and necrosis signaling pathways. Prolonged exposure to high levels of Mn ions can lead to Mn poisoning, which has symptoms that are very similar to PD, including movement disorders and muscle stiffness. Therefore, Mn ions are thought to be a possible cause of some cases of PD. The imbalance of Mn homeostasis may cause neurodegenerative diseases [14].

Gene expression analysis is becoming increasingly important in biological research. Several studies have utilized gene expression datasets downloaded from the Gene Expression Omnibus (GEO) database (https://www.ncbi.nlm.nih.gov/geo/, accessed on 27 May 2023) [15] to elucidate the biological mechanisms in the development of PD. The results of these bioinformatics analyses provide promising hints for understanding the molecular mechanisms underlying the pathogenesis of PD from different perspectives. However, no bioinformatics studies have focused on which ferroptosis-related genes (FRGs) and mitochondrial dysfunction-related genes (MDRGs) are critical for PD development. Therefore, in this study, weighted gene coexpression network analysis (WGCNA) was used to identify differentially expressed ferroptosis-related genes (DEFRGs) from 368 ferroptosis-related genes (FRGs) and PD patients from the GEO database. The DEFRGs with diagnostic values were analyzed by the receiver operating characteristic (ROC) curve (area under the curve, AUC > 0.700). Finally, the predicted protein-binding pocket of the DEFRGs was analyzed, and the best docking position was analyzed to provide a basis for new drug development.

## 2. Results

### 2.1. Identification of Differential Genes in PD

**Figure 1:**
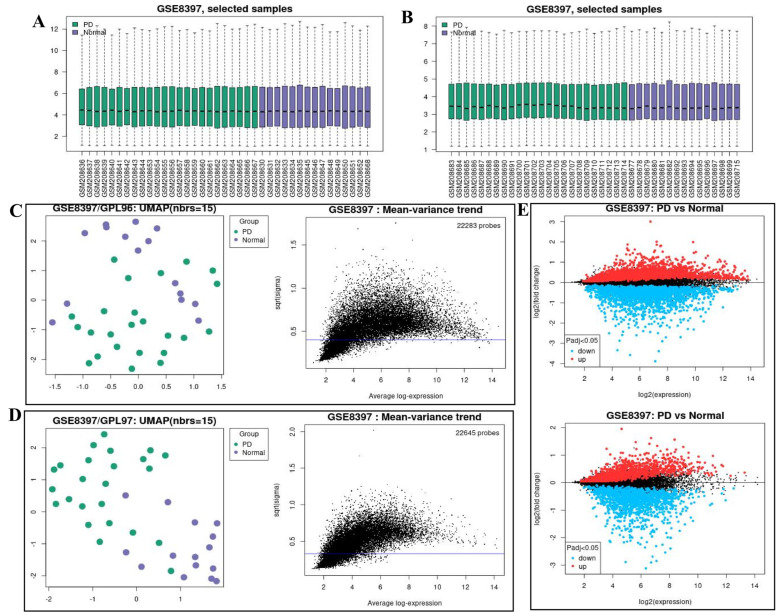
Gene chip data information. (**A**) PD sample information from the GPL96 platform in the GSE8397 dataset. (**B**) Sample information from the GPL96 platform in the GSE8397 dataset. (**C**) PCA and mean-variance trend in PD compared with the normal controls of GPL96 platform. The blue line is constant variance approximation. (**D**) PCA and mean-variance trend in PD compared with the normal controls of GPL97 platform. The blue line is constant variance approximation. (**E**) Volcano map of differential genes. Blue represents low expression and red represents high expression. *p*-value < 0.05.

The expression matrix of the GSE8397 dataset was normalized with samples from the GPL96 and GPL97 platforms (Figure 1A,B). The principal component analysis (PCA) and mean-variance trend revealed that the data were repeatable and trendless (Figure 1C,D). Volcano plots were used to visualize differential genes, and we used the thresholds of an adjusted *p*-value < 0.05 (Figure 1E and Figure S1A,B). A total of 4212 differential genes from the GPL96 platform and 2497 differential genes from the GPL97 platform were used. In the GPL96 and GPL97 platforms, the top five up- and down-regulated genes were marked in the difference ranking plot (Supplementary Figure S1C,D). The GPL96 platform included down-regulated genes 205311_at (dopa decarboxylase, *DDC*), 205857_at (solute carrier family 18 member A2, *SLC18A2*), 209560_s_at (delta-like noncanonical notch ligand 1, *DLK1*), 206836_at (solute carrier family 6 member 3, *SLC6A3*), and 208291_s_at (tyrosine hydroxylase, *TH*) and up-regulated genes 203645_s_at (CD163 molecule, *CD163*), 207526_s_at (interleukin 1 receptor like 1, *IL1RL1*), 200800_s_at (heat shock protein family a member 1A, *HSPA1*), 209309_at (alpha-2-glycoprotein 1, *AZGP1*), and 213479_at (neuronal pentraxin 2, *NPTX2*). The GPL97 platform included down-regulated genes 239913_at (*SLC10A4*), 223810_at (kelch-like family member 1, *KLHL1*), 235230_at (phosphatidylinositol specific phospholipase c x domain containing 2, *PLCXD2*), 243139_at (synaptic vesicle glycoprotein 2C, *SV2C*), and 232411_at (potassium inwardly rectifying channel subfamily j member 6, *KCNJ6*) and up-regulated genes 230863_at (LDL receptor-related protein 2, *LRP2*), 227062_at (nuclear paraspeckle assembly transcript 1, *NEAT1*), 225207_at (pyruvate dehydrogenase kinase 4, *PDK4*), 224856_at (FKBP prolyl isomerase 5, *FKBP5*), and 232054_at (protocadherin 20, *PCDH20*).

### 2.2. Immune Infiltration Analysis and GSEA

**Figure 2:**
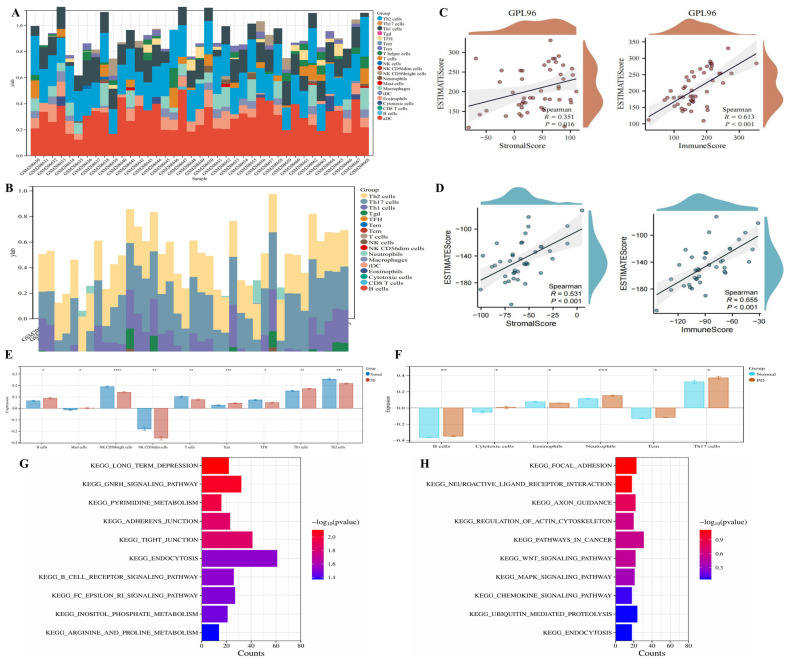
Immune infiltration analysis and GSEA of differential genes. (**A**) Heatmap of immune infiltration analysis in the GPL96 platform. (**B**) Heatmap of immune infiltration analysis in the GPL97 platform. (**C**) The correlation between the estimated scores and the stromal scores or immune scores in the GPL96 platform. (**D**) The correlation between the estimated scores and the stromal scores or immune scores in the GPL97 platform. (**E**) Boxplot of differentially expressed immune cells in the GPL96 platform. (**F**) Boxplot of differentially expressed immune cells in the GPL97 platform. (**G**) GSEA enrichment analysis of the GPL96 platform. (**H**) GSEA enrichment analysis of the GPL97 platform. Compared with normal controls, * *p* < 0.05, ** *p* < 0.01, *** *p* < 0.001, **** *p* < 0.0001.

Heatmaps of the GPL96 and GPL97 platforms of immune infiltration are shown in Figure 2A,B and showed differences in 24 markers of immune cells (Supplementary Figure S2A,B). We also analyzed the correlation between the estimated scores and the stromal scores or immune scores. According to the results, the estimated scores were positively correlated with the stromal scores or immune scores in the GPL96 and GPL97 platforms (Figure 2C,D). Compared with the normal controls from the GPL96 platform, B cells, NK CD56 dim cells, Tcm, and Th1 cells were highly expressed in PD, while Mast cells, NK CD56 bright cells, T cells, TFH, and Th2 cells were lowly expressed (Figure 2E). Compared with the normal controls from the GPL97 platform, Cytotoxic cells, Neutrophils, and Th17 cells were highly expressed in PD, while B cells, Eosinophils, and Tcm were lowly expressed (Figure 2F). In the GPL96 platform, the GSEA showed that differential genes were mainly involved in the GnRH signaling pathway, pyrimidine metabolism, adherens junction, endocytosis, B cell receptor signaling pathway, inositol phosphate metabolism, and arginine and proline metabolism (Figure 2G). In the GPL97 platform, the GSEA showed that differential genes were mainly involved in the ubiquitin-mediated proteolysis, focal adhesion, axon guidance, Wnt signaling pathway, MAPK signaling pathway, neuroactive ligand receptor interaction, chemokine signaling pathway, and endocytosis (Figure 2H).

### 2.3. WGCNA Identification of DEGs in PD

**Figure 3:**
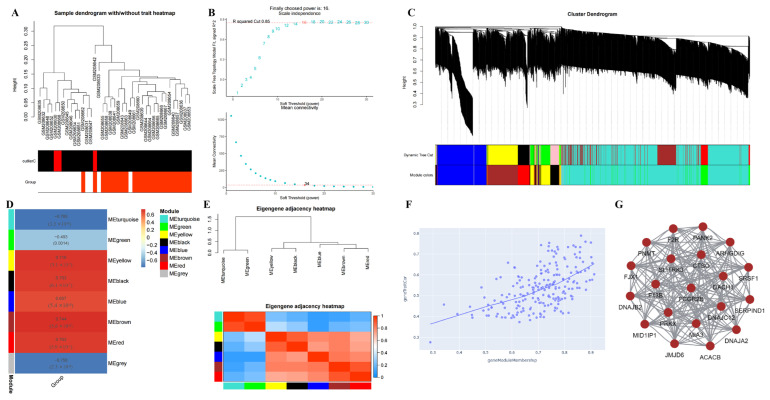
WGCNA analysis of GPL96 platform. (**A**) Cluster analysis of samples on GPL96 platform. (**B**) Scale independence and soft threshold of GPL96 platform, we chose power = 16. The ordinate represents the average connectivity number of all nodes, and most nodes have a low connectivity, so the lower the average connectivity number, the better. (**C**) Cluster analysis of genes on GPL96 platform. (**D**) Eight modules were obtained by gene clustering, and the correlation of the modules was analyzed. (**E**) Eigengene adjacency heatmap. (**F**) Relationship between gene traits and gene module members, the scatterplot of gene traits in brown module was positively correlated with gene module members. (**G**) PPI analysis of hub genes in the Brown module.

GPL96 removed duplicate genes and screened 4212 DEGs (*p* < 0.05). A sample dendrogram with/without a trait heatmap was used for cluster analysis (Figure 3A). The soft threshold represents the value of the soft threshold (power) and the Scale Free Topology Model Fit, signed R^2^ = 0.85; the higher the value of the power, the closer it is to the distribution of a scale-free network, and the more reliable is the result. In this study, we chose power = 16. The ordinate represents the average connectivity number of all nodes, and most nodes have a low connectivity, so the lower the average connectivity number, the better (Figure 3B). Cluster Dendrogram: Based on the best soft threshold and expression profile data, the upper half is the hierarchical cluster dendrogram of genes, and the lower half is the gene modules, and the genes that are closer to each other are divided into a module. The height generally represents the distance between the observed value and the cluster, and the Dynamic Tree is a dynamic module corresponding to the height (Figure 3C). Cluster analysis was performed on the eight modules (grey module is always filtered for its meaning). The correlation and credibility of all genes in each module were calculated, and the most relevant module was selected as the core module (Brown, correlation = 0.744, *p* = 5.63 × 10^−8^) (Figure 3D,E). The scatter plots of gene traits and gene module member correlations in the brown module are shown in Figure 3F. The hub genes of the brown module include pantothenate kinase 2 (*PANK2*), *cathepsin O (CTSO*), MIA SH3 domain er export factor 3 (*MIA3*), acetyl-coa carboxylase beta (*ACACB*), dachshund family transcription factor 1 (*DACH1*), intercellular adhesion molecule 4 (*ICAM4*), etc. (Figure 3G). GPL97 removed duplicate genes and screened 2497 DEGs (*p* < 0.05). By the WGCNA algorithm, we obtained the brown module as the core module (correlation = 0.69, *p* = 1.2 × 10^−6^). The hub genes of the brown module include zinc finger protein 37b, pseudogene (*ZNF37BP*), glutamate ionotropic receptor AMPA type subunit 3 (*GRIA3*), RNA-binding motif protein 15 *(RBM15*), ankyrin repeat domain 20 family (*ANKRD20*), solute carrier family 10 member 4 (*SLC10A4*), etc. (Supplementary Figure S3).

### 2.4. Identification of DEFRGs in PD

**Figure 4:**
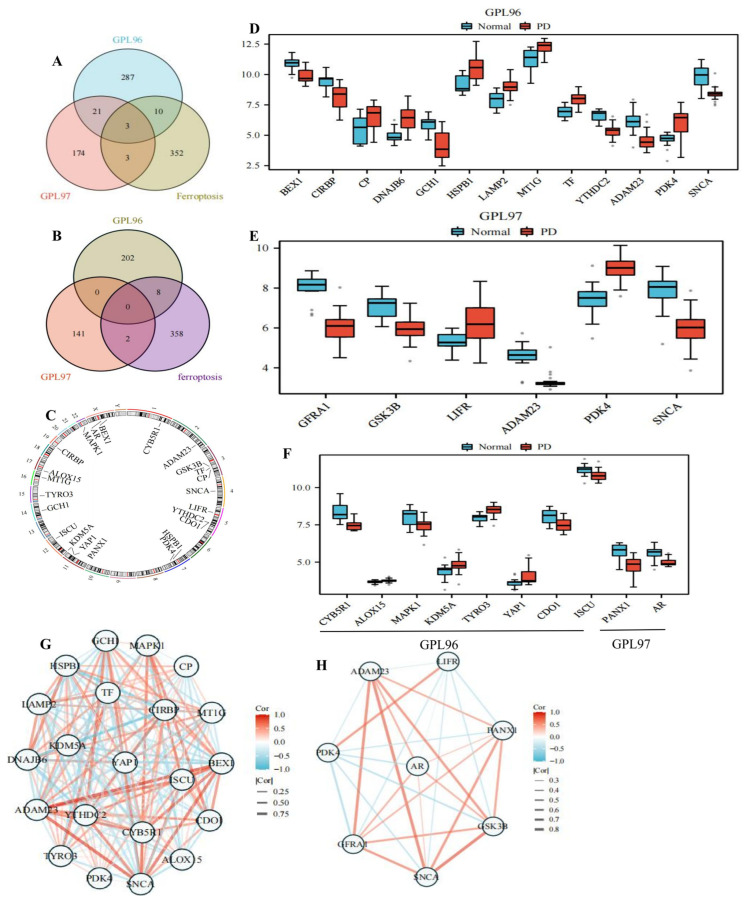
Correlation analysis of differentially expressed genes and ferroptosis-related genes. (**A**) Venn diagram of DEGs and FRGs showed that there were 3 overlapping genes between GPL96 platform, GPL97 platform, and FRGs, GPL96 and FRGs intermingled 10 genes separately, and GPL97 and FRGs intermingled 3 genes separately. (**B**) In Venn diagram, 8 genes and 2 genes were overlapping between ferroptosis and GPL96 and GPL97 platform of WGCNA, respectively. (**C**) The gene is located on the chromosome. (**D**) DEFRGs in GPL96 platform gene expression in PD compared with normal controls. (**E**) DEFRGs in GPL97 platform gene expression in PD compared with normal controls. (**F**) Gene expression differences in the GPL96 and GPL97 platform of WGCNA. (**G**) Correlation of DEFRGs in GPL96 platform. (**H**) Correlation of DEFRGs in GPL97 platform. Red represents positive correlation, and blue represents negative correlation. The stronger the inter-gene correlation, the thicker the line segment.

**Figure 5:**
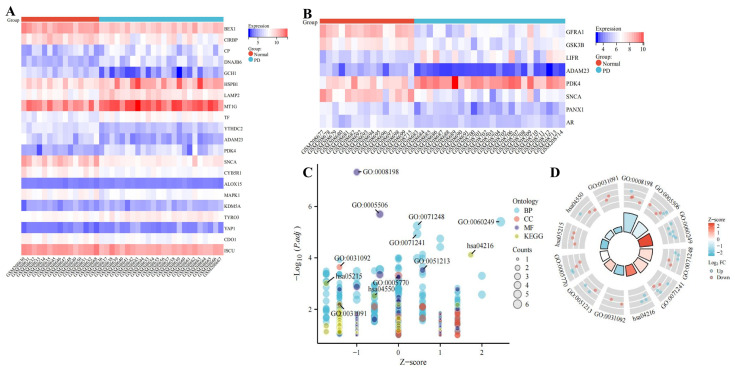
Differential gene analysis. (**A**) Gene heat map from GPL96 platform. (**B**) Gene heat map from GPL97 platform. Red represents high expression, and blue represents low expression. (**C**,**D**) Biological function and KEGG enrichment analysis, BP: GO:0060249: *HSPB1/LAMP2/PDK4/TF/TYRO3/YAP1*; GO:0071248: *MT1G/SNCA/TF/ALOX15/MAPK1*; GO:0071241: *MT1G/SNCA/TF/ALOX15/MAPK1*; CC: GO:0031092: *SNCA/CYB5R1*; GO:0005770: *LAMP2/TF/GFRA1/MAPK1*; GO:0031091: *SNCA/CYB5R1*; MF: GO:0008198: *SNCA/TF/CDO1/ISCU*; GO:0005506: *SNCA/TF/ALOX15/CDO1/ISCU*; GO:0051213: *ALOX15/KDM5A/CDO1*; KEGG: hsa04216: *CP/TF/ALOX15*; hsa05215: *GSK3B/MAPK1/AR*; hsa04550: *GSK3B/LIFR/MAPK1*.

Through screening (*p* < 0.05, |logFC| > 1), we obtained 314 genes from the GPL96 platform and 201 genes from the GPL97 platform (Supplementary Figure S2C,D). We obtained 368 FRGs through the database and used the intersection of the GPL96 and GPL97 platforms to obtain 16 DEFRGs (Figure 4A) and to obtain 10 DEFRGs of WGCNA (Figure 4B). The location of genes in the chromosome is shown in Figure 4C. Eighteen DEFRGs overlapped with the GPL96 platform, including ten up-regulated genes (*CP*, *DNAJB6*, *HSPB1*, *LAMP2*, *MT1G*, *TF*, *ALOX15*, *KDM5A*, *TYRO3*, and *YAP1*) and eight down-regulated genes (*BEX1*, *CIRBP*, *GCH1*, *YTHDC2*, *CYB5R1*, *MAPK1*, *CDO1*, and *ISCU*); five DEFRGs overlapped with the GPL97 platform, including one up-regulated gene (*LIFR*) and four down-regulated genes (*GFRA1*, *GSK3B*, *PANX1*, and *AR*); three genes overlapped with the GPL96 and GPL97 platforms, including one up-regulated gene (*PDK4*) and two down-regulated genes (*ADAM23* and *SNCA*). The differential expression heatmaps of DEFRGs in the GPL96 and GPL97 platforms’ clinical samples are shown in Figure 5A,B, and the differential expression box plots are shown in Figure 4D–F. The DEFRGs had a high correlation; red represents positive correlation, and blue represents negative correlation. The stronger the inter-gene correlation, the thicker the line segment (Figure 4G,H). For example, *BEX1* was positively correlated with *ADAM23*, *SNCA*, and *ISCU* (*p* = 0.93, *p* = 0.84, and *p* = 0.79), DNAJB6 was positively correlated with *HSPB1* (*p* = 0.81), and *CIRBP* was negatively correlated with *HSPB1*, *DNAJB6*, and *YAP1* (*p* = −0.87, *p* = −0.85, and *p* = −0.80) (Supplementary Figure S4A). GSK3B was positively correlated with *ADAM23* and *SNCA* (*p* = 0.71 and *p* = 0.84), and *PDK4* was negatively correlated with *GFRA1* and *GSK3B* (*p* = −0.61 and *p* = −0.58) (Supplementary Figure S4B).

### 2.5. Functional Enrichment Analysis of DEFRGs

The 26 genes were integrated for a gene ontology/Kyoto encyclopedia of genes and genome (GO/KEGG) enrichment analysis (Figure 5C,D). BP: anatomical structure homeostasis, cellular response to metal ion, cellular response to inorganic substance; CC: platelet alpha granule membrane, late endosome, platelet alpha granule; MF: ferrous iron binding, iron ion binding, dioxygenase activity; Pathway: ferroptosis, prostate cancer, signaling pathways regulating the pluripotency of stem cells. The physiological functions and expression locations in cells of 26 DEFRGs were retrieved from the GeneCards and human protein atlas (HPA) databases (Table 1).

### 2.6. Prognostic Analysis

**Figure 6:**
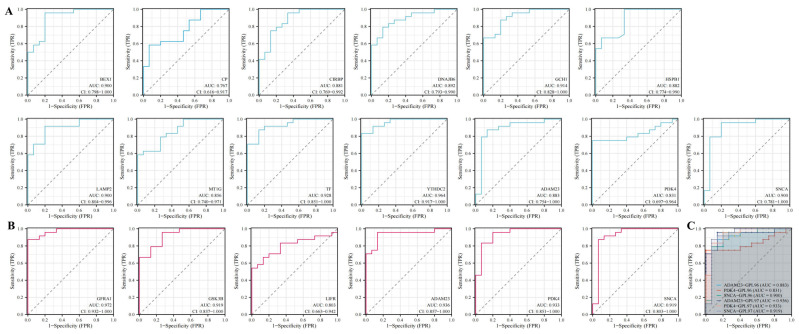
Diagnostic value of DEFRGs in (**A**) GPL96 platform, (**B**) GPL97 platform, and (**C**) combined gene analysis. ROC curves of *BEX1*, *CIRBP*, *CP*, *DNAJB6*, *GCH1*, *HSPB1*, *LAMP2*, *MT1G*, *TF*, *YTHDC2*, *GFRA1*, *GSK3B*, *LIFR*, *ADAM23*, *PDK4*, and *SNCA*.

Receiver operating characteristic curves were used to evaluate the diagnostic value of 26 DEFRGs in PD. Figure 6 and Figure S5 show the diagnostic values of the 26 hub genes in the GPL96 and GPL97 platforms. The ROC analysis showed the respective area under the curves (AUCs) of 26 hub genes. In the GPL96 platform, *BEX1* (AUC = 0.900), *GCH1* (AUC = 0.914), *LAMP2* (AUC = 0.900), *TF* (AUC = 0.928), *YTHDC2* (AUC = 0.964), *SNCA* (AUC = 0.900), and *CYB5R1* (AUC = 0.914) had very high diagnostic values. In the GPL97 platform, *GFRA1* (AUC = 0.972), *GSK3B* (AUC = 0.919), *ADAM23* (AUC = 0.936), *PDK4* (AUC = 0.933), *SNCA* (AUC = 0.919), and *PANX1* (AUC = 0.900) had very high diagnostic values.

### 2.7. Single-Cell Sequencing Analysis of DEFRGs 

**Figure 7:**
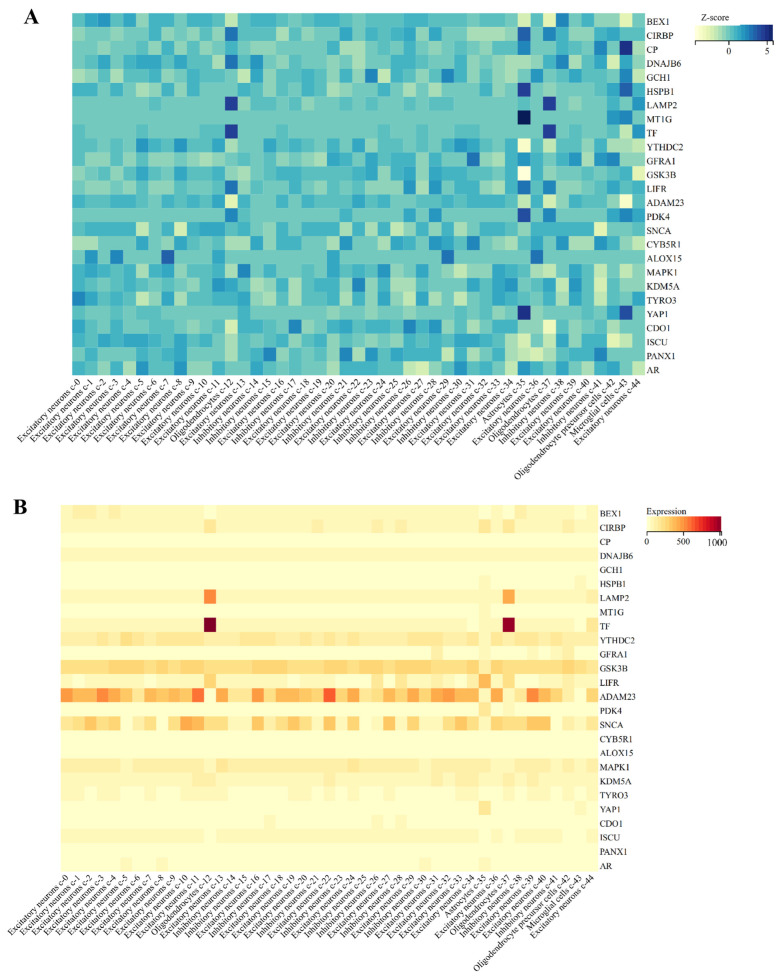
Single-cell sequencing analysis of DEFRGs. (**A**) Single-cell sequencing was used to analyze the expression of DEFRGs in the brain. (**B**) Expression Z-scores of DEFRGs in 44 cells in the brain.

We also detected the expression of DEFRGs in the brain, and found that they were enriched in different neuronal cells, oligodendrocytes, astrocytes, oligodendrocyte precursor cells, and microglial cells, and their expression scores were positively correlated (Figure 7A), e.g., *TF* and *LAMP2* are highly expressed in oligodendrocytes; *ADAM23* and *SNCA* are highly expressed in neuronal cells; LIFR is highly expressed in astrocytes; and *YTHDC2* and *GSK3B* are highly expressed in microglial cells (Figure 7B). The wide distribution of protein expressions indicates that they can be used as potential biomarkers for the treatment of PD.

### 2.8. Construction of the miRNA–Gene Network

**Figure 8:**
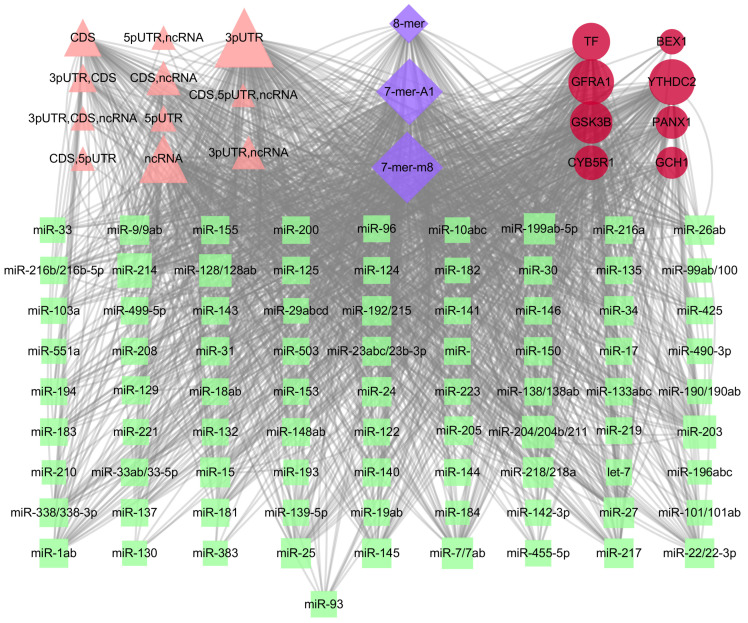
Coexpression network of diagnostic genes and target miRNAs. Red ellipses represent diagnostic genes, green quadrangles represent target miRNAs, pink triangle represent transcript regions, and blue quadrangles represent seed types.

MicroRNA (miRNA) is closely related to the occurrence and development of neurodegenerative diseases and is abnormally expressed in a variety of neurodegenerative diseases. Controlling the expression of neurodegenerative diseases-related miRNAs is expected to become a new generation of drug models for the treatment of advanced neurodegenerative diseases. Therefore, according to the area under the prognostic ROC curve, we selected genes with an AUC curve ≥ 0.9, which were used to predict miRNA, including miR-214/761/3619-5p, miR-203, miR-204/204b/211, miR-128/128ab, miR-199ab-5p, etc. The seed types of miRNA were 7-mer-m8, 7-mer-A1, and 8-mer. The transcript region comprises 5pUTR, 3pUTR, CDS, and ncRNA (Figure 8). Studies have reported that miR-214 was increased in PD patients compared with controls, MPP exposure significantly reduced cell viability of differentiated PC12 cells [16], and miR-200a and miR-204 were significantly up-regulated [17]. The overexpression of miR-128 prevented 6-OHDA-mediated mitochondrial superoxide production and induced neuronal death, respectively [18]. They can be used as potential biomarkers for the diagnosis of PD. The characterization of miRNA is expected to help effectively detect and diagnose PD.

### 2.9. In Vivo Experiment Verification

**Figure 9:**
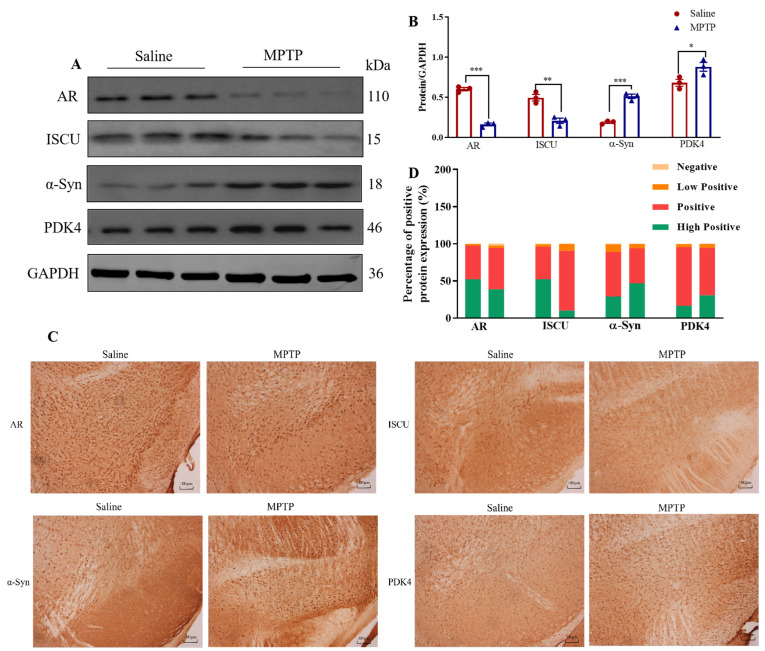
Validity verification of DEF-MDRGs. (**A**) Validation of DEF-MDRGs by Western blotting. (**B**) Statistical plots of AR, ISCU, SNCA, and PDK4. (**C**) Validation of DEF-MDRGs by immunohistochemistry. (**D**) Proportion of positive degree of differentially expressed ferroptosis–mitochondrial dysfunction-related proteins. Compared with the Saline group, * means *p* < 0.05, ** means *p* < 0.01, and *** means *p* < 0.001, n = 3.

Ferroptosis is always accompanied by mitochondrial dysfunction, and autophagy is also found to be related to the regulation of mitochondrial function in the process of biological function enrichment. We obtained 1357 mitochondrial dysfunction-related genes (MDRGs). And the intersection with 26 DEFRGs yielded 4 differentially expressed ferroptosis–mitochondrial dysfunction-related genes (DEF-MDRGs), namely, *AR*, *ISCU*, *SNCA*, and *PDK4*. In the SN of mice, compared with the Saline group, the expression of AR and ISCU was decreased (*p* < 0.05), and the expression of α-Syn and PDK4 was increased (*p* < 0.05) in the MPTP group (Figure 9A,B). The distribution of the above proteins in the SN was detected by immunohistochemistry (Figure 9C). The protein distribution was divided into High Positive, Positive, Low Positive, and Negative (Figure 9D). Compared with the Saline group, AR and ISCU High Positive decreased (*p* < 0.05), and Positive and Low Positive increased (*p* < 0.05). Inversely, for α-Syn and PDK4 protein, High Positive increased (*p* < 0.05), and Positive decreased (*p* < 0.05) (Table 2). All raw blot and gel images are available in Supplementary Figure S6. 

**Figure 10:**
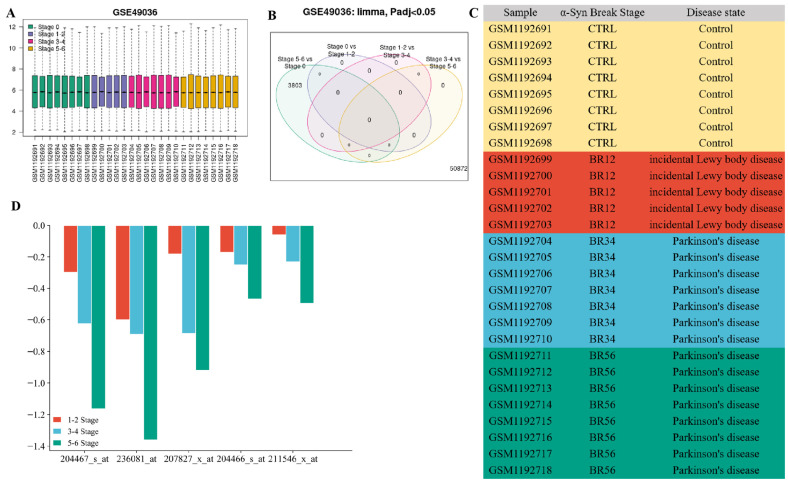
Validation of *SNCA* expression level in GSE49036 dataset (**A**) sample information of GSE49036 dataset. (**B**) A total of 3803 genes overlapped between stage 5–6 and stage 0. (**C**) The pathological stage of α-Syn in 28 samples was divided into Braak α-Syn stage 0 (Control); Braak α-Syn stage 1–2 (incidental Lewy body disease); Braak α-Syn 3–4 and Braak α-Syn stages 5–6 (PD), Orange represents control, red represents Braak α-Syn stage 1–2, blue represents Braak α-Syn 3–4, green represents Braak α-Syn stages 5–6. (**D**) Compared with stage 0, the expression differences of α-Syn in the 5 SNCA gene samples were more significant with the aggravation of pathological process, especially in stage 5–6.

We used the validation dataset GSE49036 to verify the expression changes in the SNCA gene in pathological stages (stage 0–6) again (Figure 10), which was consistent with the results of the GSE8397 dataset. Compared with stage 0, there was no difference in stage 1–2 and stage 3–4, and the expression of SNCA decreased in stage 5–6 (*p* < 0.05). This is more conducive to the process of PD. Therefore, we speculate that *SNCA* levels are negatively correlated with age, and there may also be a negative feedback regulation mechanism, where abnormal α-Syn protein degradation leads to severe protein aggregation, which negatively regulates gene levels and reduces the *SNCA* mRNA expression.

### 2.10. Prediction of Drug- and Protein-Binding Pockets

**Figure 11:**
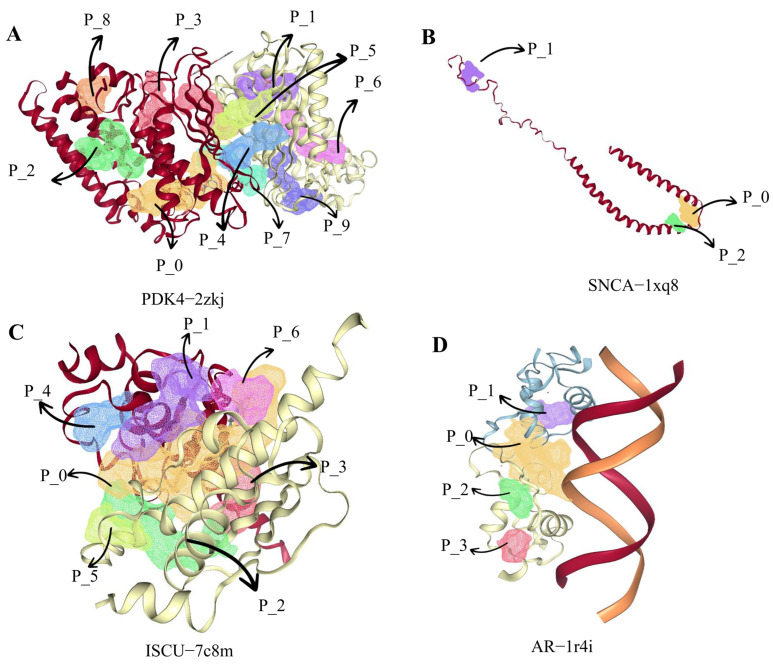
Prediction of protein-binding pockets with higher drug-binding scores, including (**A**) PDK4-2zkj, (**B**) SNCA-1xq8, (**C**) ISCU-7c8m, and (**D**) AR-1r4i.

Therapeutic drugs that target SNCA include ABBV-0805, Prasinezumab, Cinpanemab, and Gardenin A. Preclinical data show that ABBV-0805 has high selectivity for human aggregated α-Syn and very low affinity for monomers. In mouse models, treatment with murine analogs reduced α-Syn pathology and diffusion and prolonged the life span. In vitro binding of ABBV-0805 to pathological α-Syn was confirmed in postmortem brains of PD patients [19]. In the phase 2 PASADENA study on 15 September 2020, Prasinezumab did not meet its primary endpoint of slowing the progression of motor and nonmotor symptoms but showed efficacy in secondary and exploratory measures. The study showed a reduction in disease progression in all Prasinezumab-treated groups compared with placebo. The decline in motor function was reduced by 35% after one year of treatment, and patients treated for more than one year were found to have a significant delay in clinically meaningful worsening of motor progression [20]. Cinpanemab is a human monoclonal antibody against α-Syn that binds to residues 1–10 of α-Syn with an 800-fold higher affinity for aggregates than monomeric α-Syn [21]. Gardenin A is an orally potent, synthetic analogue of PMF with neurotrophic effects on neurite outgrowth and neuronal differentiation. As shown in Figure 11, protein compound binding pockets were predicted. Table 3 shows the volume, surface, and simple score of binding pockets with higher drug scores. 

## 3. Materials and Methods

### 3.1. Data Acquisition

**Figure 12:**
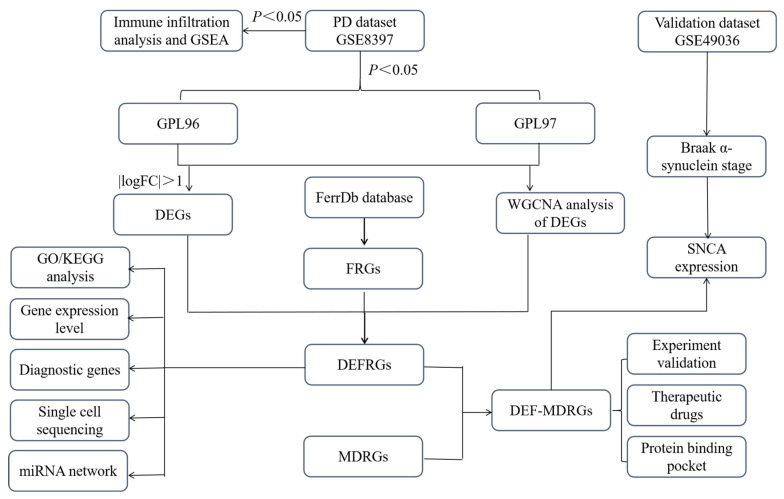
The flow chart of this study. DEGs, differentially expressed genes; FRGs, ferroptosis-related genes; DEFRGs, differentially expressed ferroptosis-related genes; GSEA, gene set enrichment analysis; GO, gene ontology; KEGG, Kyoto Encyclopedia of Genes and Genomes; MDRGs, mitochondrial dysfunction-related genes; DEF-MDRGs, differentially expressed ferroptosis–mitochondrial dysfunction-related genes.

Microarray data GSE8397 and GSE49036 were downloaded from the GEO database. GSE8397 was based on platform GPL96 [HG-U133A] Affymetrix Human Genome U133A Array and GPL97 [HG-U133B] Affymetrix Human Genome U133B Array. GSE49036 was based on platform GPL570 [HG-U133_Plus_2] Affymetrix Human Genome U133 Plus 2.0 Array. The GSE49036 dataset was divided into Control Braak α-Syn Stage 0: 8 samples; Braak α-Syn stage 1–2: 5 samples; Braak α-Syn 3–4: 7 samples; and Braak α-Syn stages 5–6: 8 samples. The GSE8397 dataset was divided into 24 PD patients and 15 normal controls. The scale function in R version 4.2.0 software was used to perform quality control and normalization of these gene expression profiles, which are represented by boxplots. PCA was used to verify the reproducibility of the data, and the R package ggord was used to construct the PCA plots. Figure 12 depicts the flow chart of this study.

### 3.2. Immune Infiltration and GSEA

The expression levels of DEFRGs between PD patients and normal controls were presented in the form of a violin plot. Immune infiltration was based on the ssGSEA algorithm provided in the R package-GSVA [22], and 24 markers of immune cells provided in the ref were used to calculate the immune infiltration of the uploaded data [23]. The expression levels of immune cells between PD patients and normal controls were presented in the form of heatmap and boxplots. Gene Set Enrichment Analysis (GSEA) was conducted using GSEA software (version 4.1.0) [24] to clarify the potential mechanism in PD. The DEGs were screened using the criteria of *p*-value < 0.05, and the enriched pathways of GSEA were screened using a *p*-value < 0.05.

### 3.3. WGCNA Identification of DEGs in PD

WGCNA aimed to find gene modules that are coexpressed and explore the association between gene networks and phenotypes of interest, as well as hub genes in the networks. We established a weighted adjacency matrix and defined a correlation power (soft thresholding parameter) showing strong relations between genes and penalizing the weak correlation. Then, we converted the adjacency into a topological overlap matrix (TOM) to measure the network connectivity of genes, and the TOM summed up the adjacent genes for the network’s gene ratio and calculated the corresponding dissimilarity. We used average linkage hierarchical clustering based on TOM dissimilarity measurement to classify genes showing similar expression profiles with gene modules, which were represented by branches and different colors of the cluster tree, constructed module relationships, and screened genes [25]. 

### 3.4. Identification of DEFRGs in PD

The “venneuler” package in R 4.2.0 software was adopted to draw the intersection of DEGs and SRGs, i.e., DESRGs. And the GSE8397 database of GPL96 and GPL97 platforms was used to verify the reliability of DESRGs, obtaining 26 DESRGs and conducting cluster analysis. The expression levels of DESRGs between PD patients and normal controls were presented in the form of scatter plots and boxplots. The R package igraph [1.4.1] and ggraph [2.1.0] were used for gene correlation analysis. After cleaning and collating the data, Spearman correlation analysis was performed on the variables in the data, and the analysis results were visualization with network diagram and using the GeneCards (https://www.genecards.org/, accessed on 1 June 2023) and HPA (https://www.proteinatlas.org/, accessed on 4 June 2023) databases to retrieve the protein biological function and distribution location. The KEGG pathway and GO enrichment of DESRGs were analyzed utilizing the “clusterProfiler” and “GOplot” packages of R (4.2.1) software. 

### 3.5. Identification of Diagnostic Genes

To screen the diagnostic genes, we visually displayed the ROC curve analysis that was performed, and the AUCs were calculated using the pROC package in R (4.2.1) software to determine the predicted values of the hub genes. Diagnostic genes were selected from the set using the criterion of AUC > 0.700.

### 3.6. Prediction of DEFRGs-Related miRNA and Protein-Binding Sites

The miRCode database (http://www.mircode.org/, accessed on 7 June 2023) was applied to predict the interaction between diagnostic genes and miRNAs, and the miRNA–gene regulatory network was visualized using Cytoscape_v3.7.2 software. The PDB database (https://www.rcsb.org/, accessed on 9 June 2023) was used to search the protein structure (PDB format). Molecular docking used the DoGSiteScorer database (https://proteins.plus/, accessed on 12 June 2023) to predict protein-binding sites.

### 3.7. In Vivo Experiment Verification

#### 3.7.1. Animal

Experiments were conducted using male C57BL/6J mice at 6–8 weeks of age and weighing 25 to 30 g. The animals were maintained in standard conditions (12/12 h light/dark cycle, 22 ± 2 °C, and relative humidity of 55 ± 5%) and allowed access to food and water ad libitum. All animal procedures conformed to the Guide for the Care and Use of Laboratory Animals and were approved by the Institutional Animal Care and Use Committee at Henan University. The experimenters were blinded to the assignments of the mice. Before the experiment, the animals were housed in our facilities for two weeks to acclimate; then, the animals were randomly divided into two groups (8 mice in each group): Saline control group and model group (MPTP, 20 mg/kg/d, intraperitoneal injection 15 days). 

#### 3.7.2. Western Blot and Immunohistochemistry Analysis

SN was homogenized with a Polytron in ice-cold RIPA buffer supplemented with PMSF (catalog#G2002 and #G2008, Servicebio, China), sonicated, and cleared by centrifugation (12,000× *g*, 10 min, at 4 °C). Protein concentration in the supernatant was determined by BCA assay and separated on SDS-PAGE gels and transferred onto nitrocellulose membrane (Millipore, IPFL00010, Germany) by electrophoresis. Blots were blocked in 5% nonfat milk in TBST for 1 h at room temperature and probed with primary antibody, including AR (1:1000, Affinity, Melbourne), ISCU (1:1000, Affinity, Melbourne), α-Syn (1:1000, Affinity, Melbourne), PDK1 (1:1000, Affinity, Melbourne), and GAPDH (1:3000, Affinity, Melbourne) in TBST with 1% nonfat milk overnight at 4 °C. After overnight incubation, this was followed by incubation with HRP-conjugated secondary antibody in TBST with 1% nonfat milk for 2 h at room temperature. The blots were developed using an Enhanced Chemiluminescence assay (BIO-Rad). Immunohistochemistry was performed on 30 μm thick serial brain sections. We used 1xCitrate Antigen Retrieval Solution, 98 °C for 10 min, 0.1% Triton X-100 transparent for 10 min, hydrogen peroxide for 20 min (dark), and blocked with 10% goat serum in PBS incubated with AR (1:200, Affinity, Melbourne), ISCU (1:200, Affinity, Melbourne), α-Syn (1:200, Affinity, Melbourne), and PDK1 (1:200, Affinity, Melbourne) antibodies overnight. Brain tissues were treated with appropriate biotin secondary antibody, followed by DAB Reagent Kit (catalog#G1212, Servicebio, China). Densitometry analysis was performed on scanned Western blot and immunohistochemistry images using the ImageJ software (https://imagej.net/software/imagej/, accessed on 14 June 2023). 

### 3.8. Statistical Analysis

R 4.2.0 software and Adobe Photoshop 2021 were employed in this study. Data are presented as the mean ± SEM, and comparisons between groups were performed using an unpaired Student’s *t*-test. ROCs were used to evaluate AUCs and predictive abilities. A *p*-value of less than 0.05 was considered statistically significant.

## 4. Conclusions

PD will continue to develop with time and age, and the disease will become more and more severe. PD first causes a decrease in dopamine in the brain, which leads to constipation and olfactory dysfunction. The pontine nerves then deteriorate, affecting the central nerves of the brain, which can lead to depression and severe insomnia. Then, the symptoms of tremors and brain nerve damage affect normal thinking. Finally, when PD is approaching the late stage, dementia symptoms occur, and at this time, the patient has begun to be confused and could be completely unable to take care of themselves [26]. Apoptosis may contribute to the extensive DA neuronal degeneration in PD. However, the generally enhanced neuroinflammation and features of chronic degeneration found at autopsy in PD patients cannot be explained by apoptosis alone [27]. During degeneration of dopaminergic neurons in PD, several phenomena that are similar to the characteristic pathological changes of ferroptosis were observed, such as SN iron deposition leading to glutathione (GSH) loss, increased lipid peroxidation and ROS, and mitochondrial dysfunction. Treatment with N-acetylcysteine (NAC), a precursor of GSH, reduced the in vivo neuronal damage caused by PD [28]. In addition, the use of iron chelators has been shown to significantly improve the motor symptoms of PD in patients (phase II clinical trials) and animal models [29]. These results suggest that the ferroptosis pathway may play a key role in the development of PD.

In this study, through different multiples |logFC| > 1 and the WGCNA algorithm, we have shown a difference between FRG intersections, with a total of 26 DEFRGs, and we detected the expression of DEFRGs in the brain, and found that they were enriched in different neuronal cells, oligodendrocytes, astrocytes, oligodendrocyte precursor cells, and microglial cells, and their expression scores were positively correlated. Different from the classical apoptosis, the ferroptosis process has no cell shrinkage, chromatin condensation, and other phenomena, but there is mitochondrial shrinkage and increased lipid peroxidation. Therefore, we interlaced the MDRGs to obtain four hub genes, namely, *AR*, *ISCU*, *SNCA*, and *PDK4*. At the same time, a large number of studies have shown that the above genes play a key role in ferroptosis and PD [30,31].

Researchers have identified the mechanisms of action of four iron–sulfur clusters: nitrogen fixation (NIF), the iron–sulfur cluster (ISC), sulfur mobilization (SUF), and the cytosolic iron–sulfur cluster assembly (CIA). The synthesis of ISC is critical for cell survival, especially mitochondrial activity and function. Sporadic PD, which accounts for more than 90% of PD cases, is caused by the blockage of pathways that regulate the power source of nerve cells, mitochondria. The blockage leads to the accumulation of large amounts of damaged mitochondria that fail to generate sufficient energy for the cells, which in turn leads to progressive neuronal death and, ultimately, PD symptoms [32]. The overexpression of ISCU (a mitochondrial protein) significantly attenuated dihydroartemisinin-induced ferroptosis by regulating iron metabolism, rescuing the mitochondrial function and increasing the level of GSH [33]. The inhibition of the ISCU expression reduced free adenosine 5′-triphosphate (ATP) levels, whereas the overexpression of ISCU reversed ATP levels. Consistent with our study, the pathogenic role of an ISCU expression reduction in PD is found for the first time, which also points us to the development of future therapeutic drugs for PD against ISCU targets.

The serum testosterone (T) concentration gradually decreases after 30 years of age in men, and this decrease is associated with the occurrence and development of age-related neurodegenerative diseases such as Alzheimer’s disease (AD) and PD. The incidence of PD in elderly male patients is significantly higher than that in elderly female patients. The symptoms of male AD patients can be exacerbated by a T deficiency, implicating a potential role of T in neurodegenerative diseases. In the clinical observation of androgen deprivation therapy (ADT) for prostate cancer, it is also indirectly suggested that androgen decline may increase the risk of AD [34]. Although studies have shown that T supplementation improves the cognitive function and alleviates motor and nonmotor symptoms of PD in male AD patients, it is not known whether T improves mitochondrial function during brain aging. In our study, AR belongs to the mitochondrial dysfunction gene, and its reduction may aggravate the pathogenesis of PD. T can regulate neuronal growth, differentiation, survival, or death through both genomic and nongenomic signaling pathways. The classic genomic pathway involves AR binding to specific DNA response elements in the promoters of target genes and regulating mRNA transcription and protein synthesis. It has been shown that physiological concentrations of T can directly induce neuroprotective effects via AR in primary cultured neurons, and that AR regulates TH transcriptional activity [35]. In mouse myoblasts, H_2_O_2_-induced oxidative stress decreases the expression of mitochondrial genes, while T can increase the expression of mitochondrial genes and nuclear-encoded mitochondrial biogenesis-related genes, and it may directly regulate mitochondrial transcription through AR. Therefore, T may regulate the expression of mitochondria-related genes through AR and improve mitochondrial dysfunction during brain aging [36].

The *SNCA* encodes an α-Syn protein, a family that also includes β-Syn and γ-Syn. α-Syn is abundantly expressed in brain tissue. The protein can selectively inhibit phospholipase D2 and bind to calcium channels. α-Syn is also involved in the integration of presynaptic signals and membrane transport, and its specific functions are to regulate synaptic vesicle transport and control the release of neurotransmitters from vesicles. However, α-Syn can also form plaques (Lewy bodies) due to the abnormal aggregation of α-Syn itself or the brain environment, which affects the normal function of neurons and brain tissues. The defects of this gene are closely related to the pathogenesis of PD. α-Syn has been functionally linked with the metabolism of both iron and lipid, suggesting a possible interplay between dysregulated α-Syn and other PD pathological hallmarks that are related to ferroptosis [37]. In our study, a low mRNA expression and high protein expression may lead to a compensatory decrease in the *SNCA* expression due to an increased α-Syn protein accumulation in somatic cells, and PD is characterized by the perinuclear accumulation of aggregated α-Syn. The increase in these proteins can act as a negative feedback mechanism to decrease SNCA gene expression. Alternatively, a reduced *SNCA* expression may simply be a normal aging process. α-Syn mRNA levels are reduced in normally aging humans and rodents. Thus, since aging is a major risk factor for PD, a reduced *SNCA* expression may represent an independent comorbidity that does not actually contribute to the pathogenesis of sporadic PD [38]. That being said, while changes in the *SNCA* expression may not directly contribute to neurotoxicity in sporadic PD, it is clear that the shift from soluble to insoluble α-Syn protein levels is directly related to PD pathogenesis.

PDK4 is the top gene that is responsible for ferroptosis resistance. PDK4 inhibits ferroptosis by blocking pyruvate dehydrogen-dependent pyruvate oxidation [39]. PDK2/4 deficiency suppressed the diabetes-induced lactate surge, pain-related ion channel expression, satellite glial cell activation, and macrophage infiltration. In addition, PDK2/4 deficiency also reduced the central sensitization and neuroinflammatory features in the spinal cord, which may be responsible for the attenuated pain hypersensitivity [40]. PDK-mediated phosphorylation leads to a metabolic shift toward glycolysis and the production of lactate as the end product. Although lactate is the most important energy source for neurons, increased lactate accumulation or lactic acidosis is associated with neuronal damage [41]. Glia and macrophages are also sources of lactic acid under diverse pathological conditions [40,42]. A lactic acid-induced acidic microenvironment provokes the activation of glial cells and macrophages and thereby favors the release of diverse proinflammatory mediators, suggesting that the decreased pH due to accumulation of lactic acid might serve as a potential inducer of proinflammatory cytokine production. Pyruvate dehydrogenase kinases (PDKs) are mitochondrial enzymes that suppress the conversion of pyruvate to acetyl CoA via inhibitory phosphorylation of the pyruvate dehydrogenase complex (PDC). PDC enters mitochondria and increases their metabolic activity, thereby providing more ATP for cells and inhibiting the development of neurodegenerative diseases. This finding opens a new perspective for the treatment of PD. Decreased cellular energy production is a characteristic of PD, and age-related decline in cellular energy production is a major risk factor for PD. However, further studies are needed to explore the specific mechanisms for more precise improvement of PD-related symptoms. Although the four diagnostic genes we screened have been reported to be associated with ferroptosis and mitochondrial dysfunction, there are not enough studies to confirm the role of a ferroptosis pathway that is regulated by these genes in PD. Therefore, more in vitro and in vivo studies are needed to confirm these findings. This is a limitation of this study.

In conclusion, understanding the effects of ferroptosis on PD can be useful for the prevention of the disease, diagnosis, and treatment by providing new ideas and methods. This may include an intervention in the iron metabolism to prevent the liberation and deposition of iron ions and drug therapy to inhibit oxidative stress and inflammatory responses. AR, ISCU, SNCA, and PDK4 have the potential to be specific biomarkers for the early diagnosis of PD. By investigating the mechanisms and effects of ferroptosis, we can better understand the progression of PD and provide important scientific evidence for the development of new therapeutic strategies.

## Figures and Tables

**Table 1:** The information for the 26 DEFRGs.

Gene	Name	Biological Function	Expression Variance	Main Location
*BEX1*	Brain-Expressed X-Linked Protein 1	BEX1 is involved in the regulation of neuronal differentiation and apoptosis through the P75 neurotrophin receptor pathway.	Down	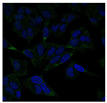
*CIRBP*	Cold-Inducible RNA-Binding Protein	CIRBP regulates cell proliferation, development, apoptosis, differentiation, biological rhythm regulation, and inflammation.	Down	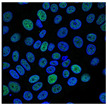
*CP*	Ceruloplasmin	It exhibits ferroxidase activity and oxidizes Fe^2+^ to Fe^3+^ without releasing free radical oxygen.	Up	-
*DNAJB6*	DnaJ Heat Shock Protein Family (HSP40) Member B6	Having stimulatory effects on the atpase activity of HSP70 in a dose-dependent and time-dependent manner and therefore acting as cochaperones of HSP70, members of this family play a specific role in neuronal aggregation of polyglutamine.	Down	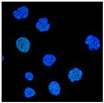
*GCH1*	GTP Cyclohydrolase 1	GCH1 is the most important rate-limiting enzyme in BH_4_ synthesis, and the decrease in GCH1 activity leads to a decrease in BH_4_ production. It is also a major regulator of peripheral neuropathic and inflammatory pain, and gene mutations may reduce pain sensitivity. It is involved in dopamine synthesis.	Down	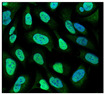
*HSPB1*	Heat shock protein family B	It is transported from the cytoplasm to the nucleus upon stress induction and acts as a molecular chaperone to promote the proper folding of other proteins. It can regulate many biological processes through its molecular chaperone activity, including phosphorylation of neurofilament proteins and axonal transport.	Up	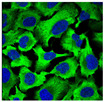
*LAMP2*	Lysosomal-Associated Membrane Protein 2	LAMP2 plays a role in lysosomal protection, maintenance, and adhesion, regulating chaperone-mediated autophagy.	Up	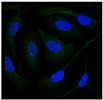
*MT1G*	Metallothionein 1G	MT1G is involved in the response of cells to metal ions. Cellular response to vascular endothelial growth factor stimulation, negative regulation of growth.	Up	-
*TF*	Transferrin	Responsible for transporting iron from sites of absorption and heme degradation to sites of storage and utilization, stimulating cell proliferation.	Up	-
*YTHDC2*	YTH N6-Methyladenosine RNA Binding Protein C2	RNA helicases play a role in the efficiency and stability of RNA processing by specifically recognizing and binding N6-methyladenosine (m6A)-containing RNA by facilitating the transition from mitosis to meiosis in stem cells.	Down	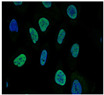
*GFRA1*	Glial cell line-derived neurotrophic factor Family Receptor Alpha 1	GFRA1 encodes a member of the glial cell line-derived neurotrophic factor receptor (GDNFR) family of proteins. Glial cell line-derived neurotrophic factor (GDNF) and neurotrophin (NTN) are two structurally related potent neurotrophic factors that play a key role in controlling neuronal survival and differentiation.	Down	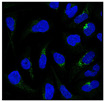
*GSK3B*	Glycogen Synthase Kinase 3 Beta	Negative regulator of glucose homeostasis involved in energy metabolism, inflammation, endoplasmic reticulum stress, mitochondrial dysfunction, and apoptotic pathways. GSK3B deficiency has been implicated in PD and Alzheimer’s disease.	Down	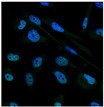
*LIFR*	LIF Receptor Subunit Alpha	LIFR encodes a protein belonging to the type I cytokine receptor family that mediates the action of leukemia-inhibitory factors, which are involved in cell differentiation, proliferation, and survival in adults and embryos.	Up	-
*ADAM23*	ADAM Metallopeptidase Domain 23	Members of this family are membrane-anchored proteins that are structurally related to snake venom disintegrin and are implicated in various biological processes involving cell–cell and cell–matrix interactions, including fertilization, muscle development, and neurogenesis.	Down	-
*PDK4*	Pyruvate Dehydrogenase Kinase 4	The activity of pyruvate dehydrogenase complex (PDC) is inactivated and tricarboxylic acid cycle and lipid biosynthesis are blocked.	Up	-
*SNCA*	Alpha-Synuclein	Regulates synaptic vesicle trafficking and subsequent neurotransmitter release. SNCA can be used to integrate presynaptic signaling and membrane trafficking. SNCA deficiency has been implicated in the pathogenesis of PD.	Down	-
*CYB5R1*	Cytochrome B5 Reductase 1	REDOX proteins catalyze the peroxidation of lipids containing long-chain phospholipids of unsaturated fatty acids, causing oxidative damage and leakage of liposome membranes. They can transfer electrons from NADPH/NADH to downstream proteins to mediate REDOX reactions.	Down	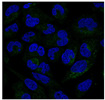
*ALOX15*	Arachidonate 15-Lipoxygenase	ALOX15 encodes a member of the lipoxygenase protein family. The encoded enzymes act on various polyunsaturated fatty acid substrates to produce various bioactive lipid mediators that can regulate inflammation and immunity.	Up	-
*MAPK1*	Mitogen-Activated Protein Kinase 1	It serves as an integration point for a variety of biochemical signals and is involved in various cellular processes such as proliferation, differentiation, transcriptional regulation, and development.	Down	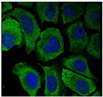
*KDM5A*	Lysine Demethylase 5A	As a histone demethylase, the encoded protein demethylates lysine 4 of histone H3 through histone coding, thus playing a role in gene regulation and closely related to tumor proliferation.	Up	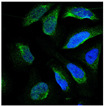
*TYRO3*	Tyrosine-Protein Kinase Receptor	TYRO3 is involved in the control of cell survival, migration and differentiation, spermatogenesis, immune regulation, and phagocytosis.	Up	-
*YAP1*	Yes1-Associated Transcriptional Regulator	Transcriptional regulators, key downstream regulatory targets in the Hippo signaling pathway, play a key role in organ size control and tumor suppression by limiting proliferation and promoting apoptosis.	Up	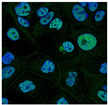
*CDO1*	Cysteine Dioxygenase Type 1	Taurine is a key enzyme that is synthesized by the organism itself. Taurine is abundant and widely distributed in the brain, which can significantly promote the growth and development of the nervous system and cell proliferation and differentiation, and it plays an important role in the development of brain nerve cells. It is involved in L-cysteine catabolism.	Down	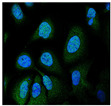
*ISCU*	Iron-Sulfur Cluster Assembly Enzyme	Enzymes that regulate metabolism, iron homeostasis, and oxidative stress responses.	Down	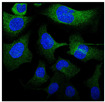
*PANX1*	Pannexin 1	Structural components of gap junctions and hemichannes, involved in ATP release and nucleotide permeation. It can act as a Ca^2+^ leakage channel to regulate endoplasmic reticulum Ca^2+^ homeostasis.	Down	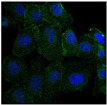
*AR*	Androgen Receptor	Ligand-activated transcription factors that regulate eukaryotic gene expression and affect cell proliferation and differentiation in target tissues, physiological concentrations of testosterone induce neuroprotective effects through AR.	Down	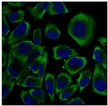

**Table 2:** Proportion of AR, ISCU, SNCA, and PDK4 positive degree ($\bar{X}$ ± SEM).

Protein Positive Degree Proportion (%)		High Positive	Positive	Low Positive	Negative
Protein					
AR	Saline	51.51 ± 0.65	45.70 ± 0.87	2.25 ± 0.11	0.54 ± 0.19
	MPTP	38.54 ± 1.12 ***	55.31 ± 1.19 **	3.21 ± 0.11 **	2.93 ± 0.37 **
ISCU	Saline	52.26 ± 1.16	43.52 ± 1.22	3.20 ± 0.10	1.02 ± 0.24
	MPTP	10.02 ± 0.29 ***	80.33 ± 0.48 ***	9.41 ± 0.34 **	0.25 ± 0.075 *
SNCA	Saline	27.60 ± 0.66	60.50 ± 0.40	10.65 ± 0.29	1.24 ± 0.28
	MPTP	47.00 ± 0.84 ***	46.26 ± 0.89 ***	6.32 ± 0.13 ***	0.42 ± 0.10 *
PDK4	Saline	16.11 ± 0.33	79.56 ± 0.47	3.75 ± 0.48	0.59 ± 0.31
	MPTP	31.07 ± 0.20 ***	63.51 ± 0.62 ***	5.01 ± 0.54	0.41 ± 0.17

Note: * means compared with Saline group * *p* < 0.05, ** *p* < 0.01, *** *p* < 0.001, n = 3.

**Table 3:** Volume, surface area, drug score, and simple score of the protein pocket.

Protein	Name	Volume Å^3^	Surface Å^2^	Drug Score	Simple Score
PDK4 (2zkj)	P_3	535.61	618.17	0.89	0.25
	P_9	301.66	393.37	0.84	0.15
	P_0	939.76	1168.93	0.83	0.64
	P_1	841.81	920.6	0.81	0.57
	P_2	551.09	639.63	0.81	0.29
	P_6	387.17	498.25	0.79	0.19
	P_7	330.28	444.89	0.71	0.06
	P_5	388.33	549.11	0.67	0.17
	P_4	392.29	612.63	0.65	0.15
	P_8	310.97	447.95	0.55	0.29
SNCA (1xq8)	P_2	130.6	321.36	0.2	0.04
	P_1	321.36	762.65	0.37	0.22
	P_0	406.65	887.92	0.55	0.32
ISCU (7c8m)	P_0	1158.91	1285.98	0.81	0.6
	P_1	383.3	674.44	0.81	0.24
	P_2	367.74	669.91	0.74	0.22
	P_3	314.82	427.98	0.52	0.23
	P_4	141.31	341.21	0.35	0.0
	P_5	140.03	305.42	0.34	0.0
	P_6	109.18	284.76	0.24	0.0
AR (1r4i)	P_0	848.19	1180.27	0.79	0.57
	P_1	130.43	290.87	0.26	0
	P_2	112.51	212.86	0.17	0
	P_3	106.56	327.51	0.22	0

## Data Availability

All data and material generated or analyzed during this study are included in this published article (and its supplementary materials files) (https://www.jianguoyun.com/p/DRDyu8QQ7uyQDBjt9aIFIAA, accessed on 14 November 2023).

## Supplementary Materials

The following supporting information can be downloaded at: https://www.mdpi.com/article/10.3390/ijms242417203/s1.
