# Supplementary material for: The Construction and Validation of a Novel Ferroptosis-Related Gene Signature in Parkinson’s Disease
Source: Int J Mol Sci. 2023 Dec 6;24(24):17203. doi: 10.3390/ijms242417203 (PMC10742934; doi:10.3390/ijms242417203)
Supplement: Supplementary file 1 [file ijms-24-17203-s001.zip › ijms-2755172-supplementary.pdf]

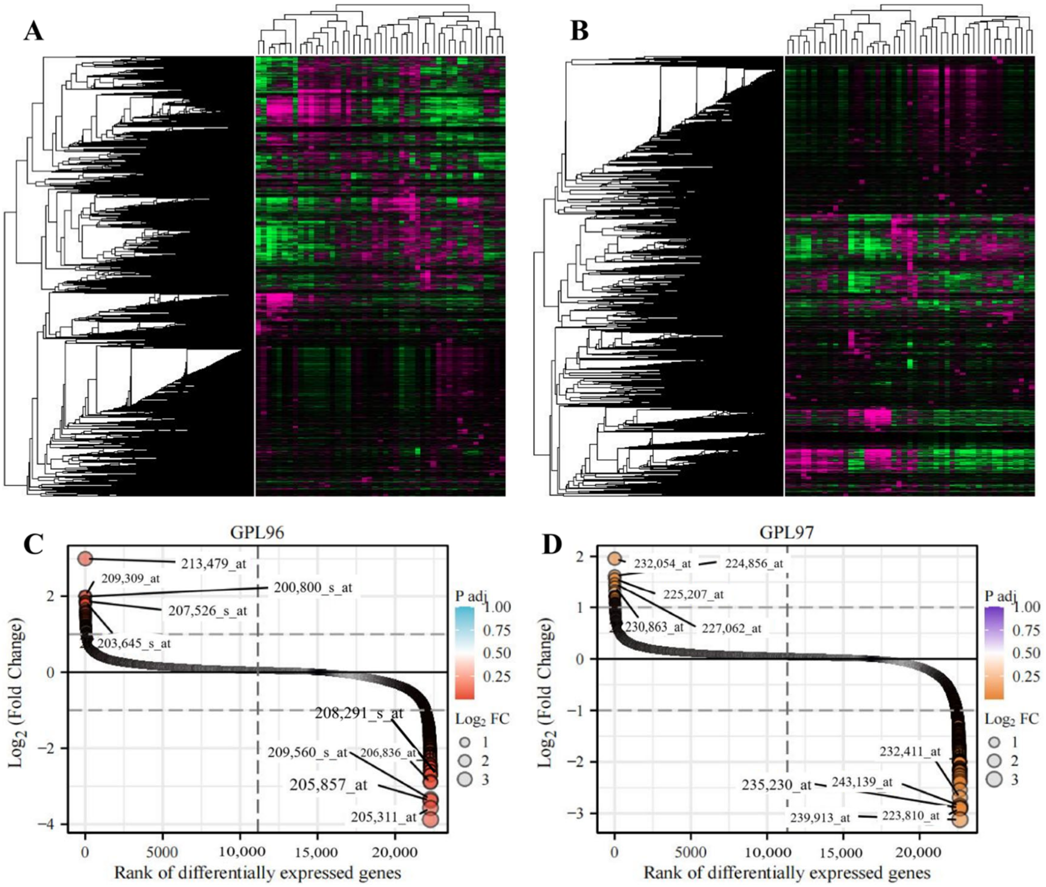

Figure S1. Differential genes in the GSE8397 dataset. (**A**) From the GPL96 platform gene expression heat map. (**B**) From the GPL97 platform gene expression heat map, pink represents high expression genes, green represents low expression genes. (**C**) The difference ranking plot of differential gene between the PD and healthy controls in the GPL96 platform. (**D**) The difference ranking plot of differential gene between the PD and healthy controls in the GPL97 platform.

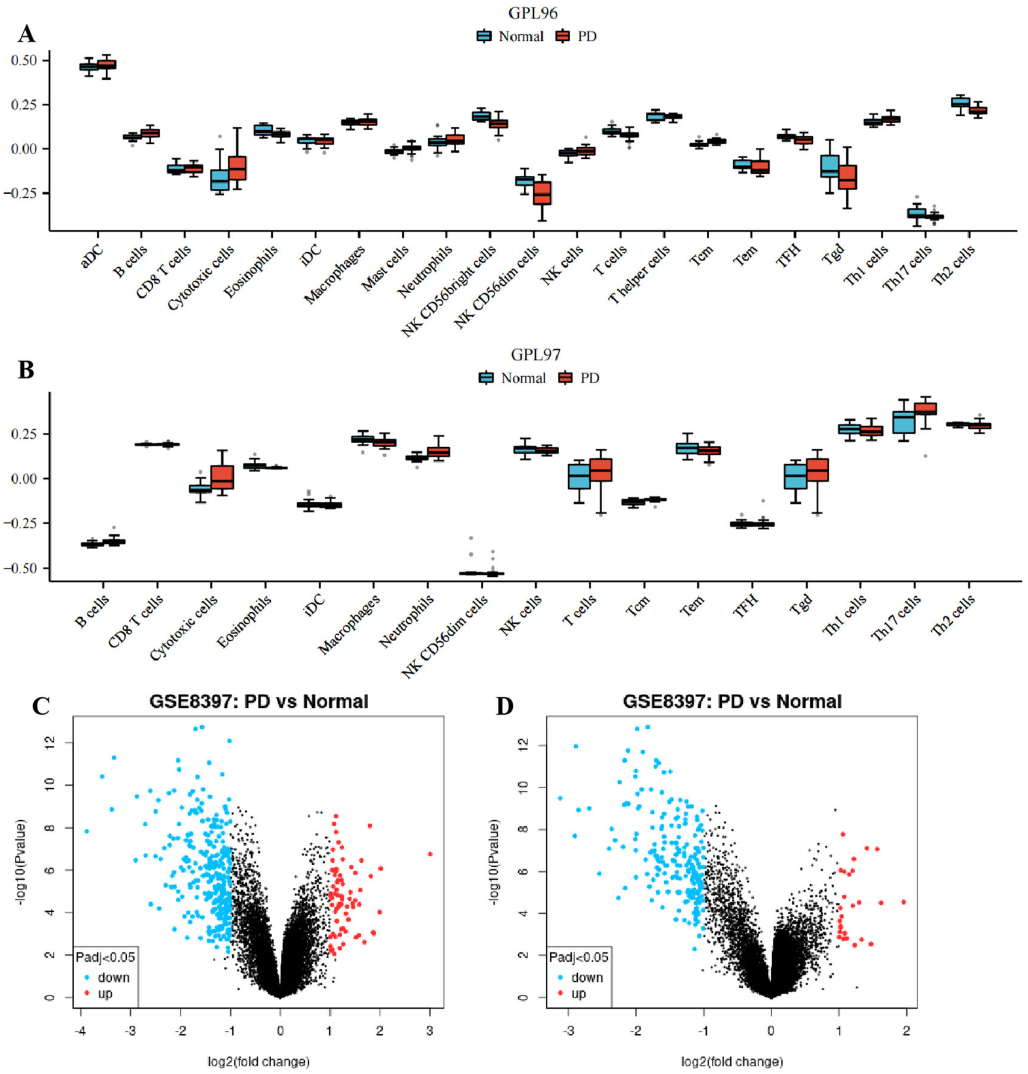

Figure S2. Boxplots of immune cell expression in the (**A**) GPL96 platform and (**B**) GPL97 platform. Heat map of differentially expressed genes on (**C**) GPL96 platform and (**D**) GPL97 platform, $p < 0.05$, $|\text{logFC}| > 1$, blue represents low expression and red represents high expression.

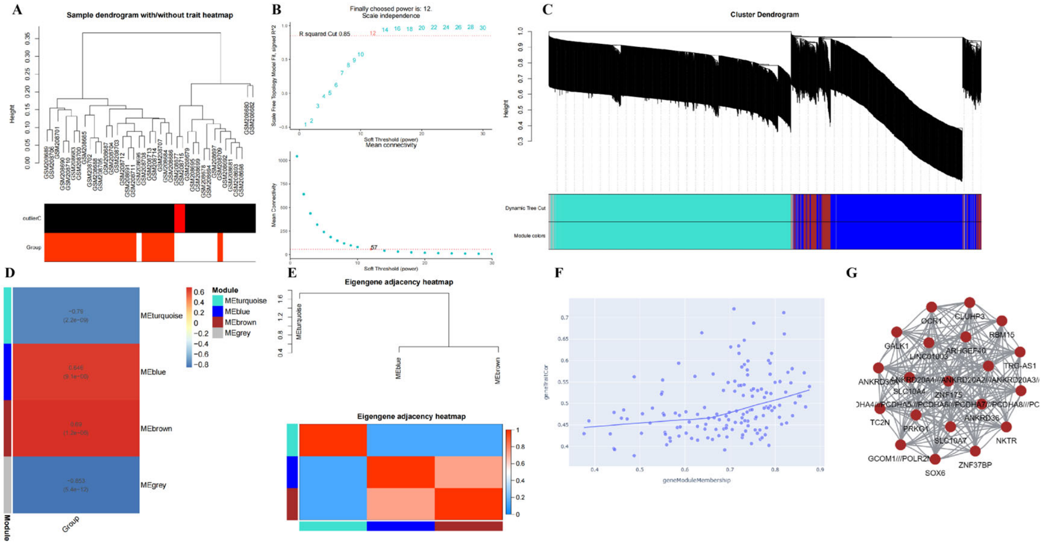

Figure S3. WGCNA analysis of GPL97 platform. (**A**) Cluster analysis of samples on GPL97 platform. (**B**) Scale independence and soft threshold of GPL97 platform. (**C**) Cluster analysis of genes on GPL97 platform. (**D**) Four modules were obtained by gene clustering, and the correlation of the modules was analyzed. (**E**) Eigengene adjacency heatmap. (**F**) Relationship between gene traits and gene module members. (**G**) PPI analysis of hub genes in the Brown module.

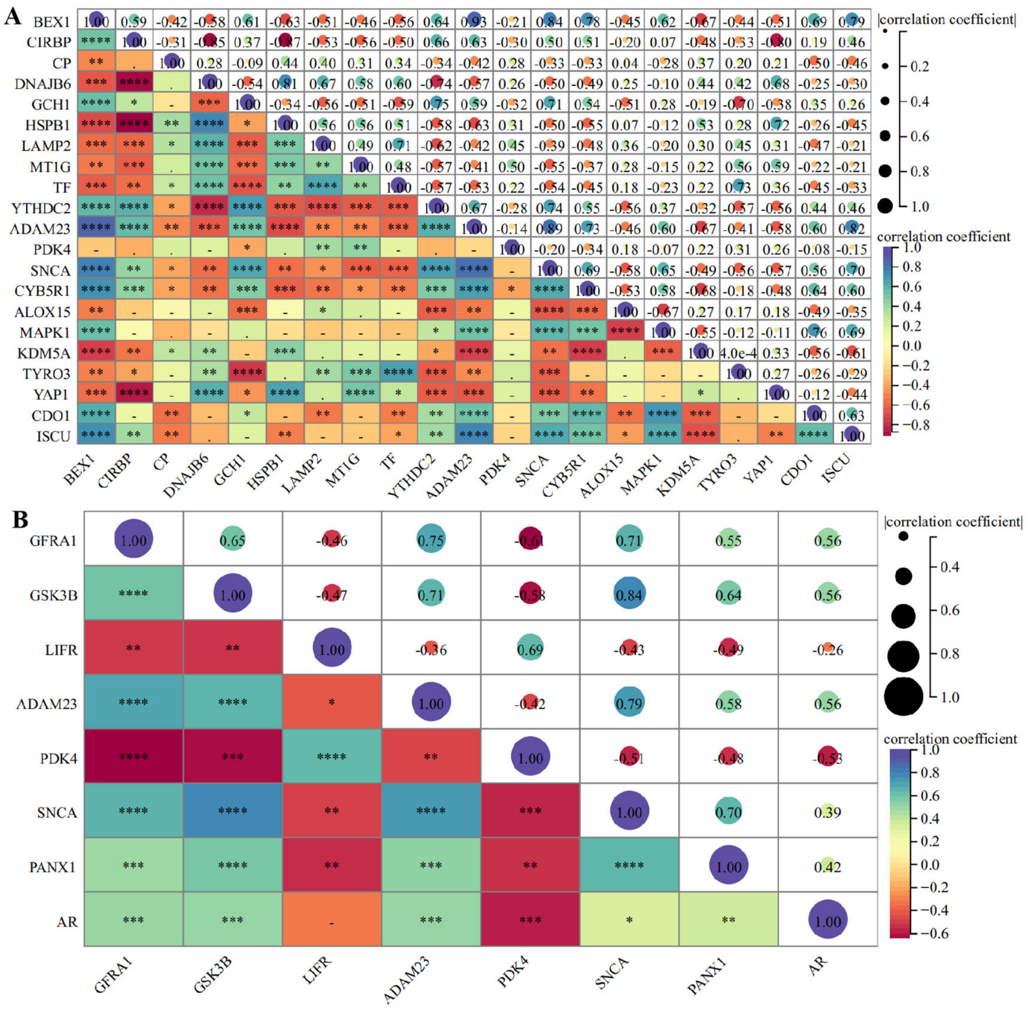

FIigure S4. Correlation of DEFRGs in (**A**) GPL96 and (**B**) GPL97 platforms. * means $p < 0.05$, ** means $p < 0.01$, *** means $p < 0.001$, and **** means $p < 0.0001$.

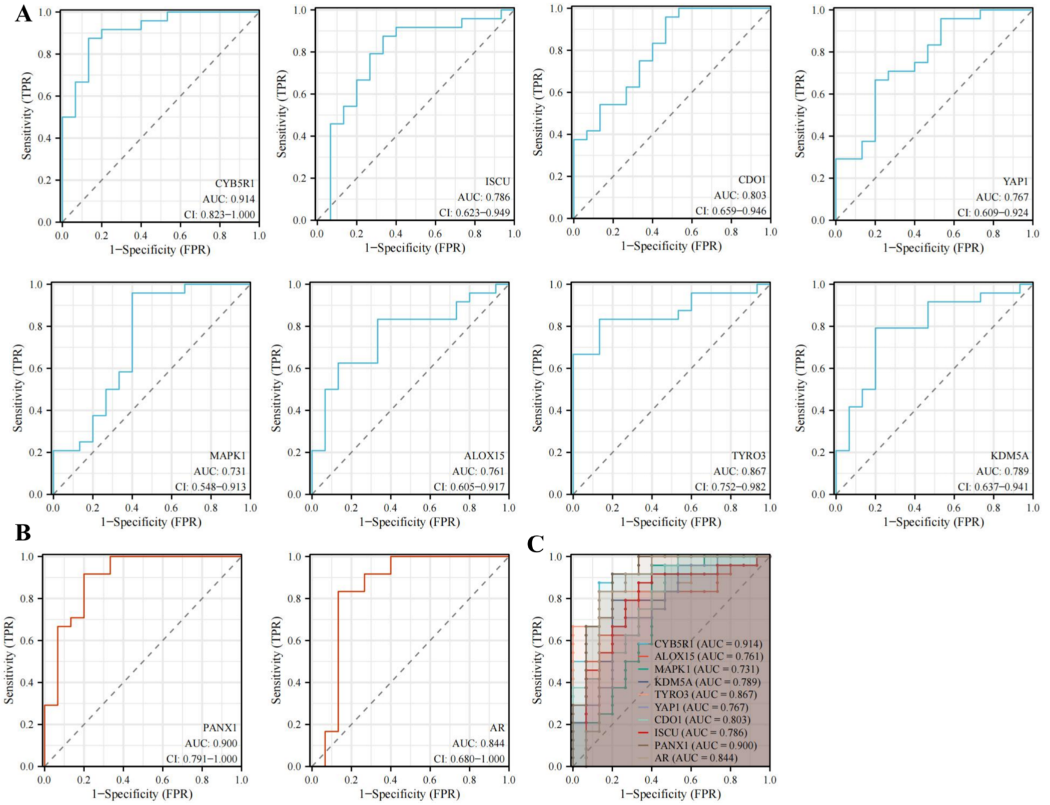

Figure S5. Diagnostic value of 10 hub genes in WGCNA of (**A**) GPL96 platform, (**B**) and GPL97 platform, and (**C**) Combined gene analysis. ROC curves of CYB5R1, ALOX15, MAPK1, KDM5A, TYRO3, YAP1, CDO1, ISCU, PANX1, and AR.

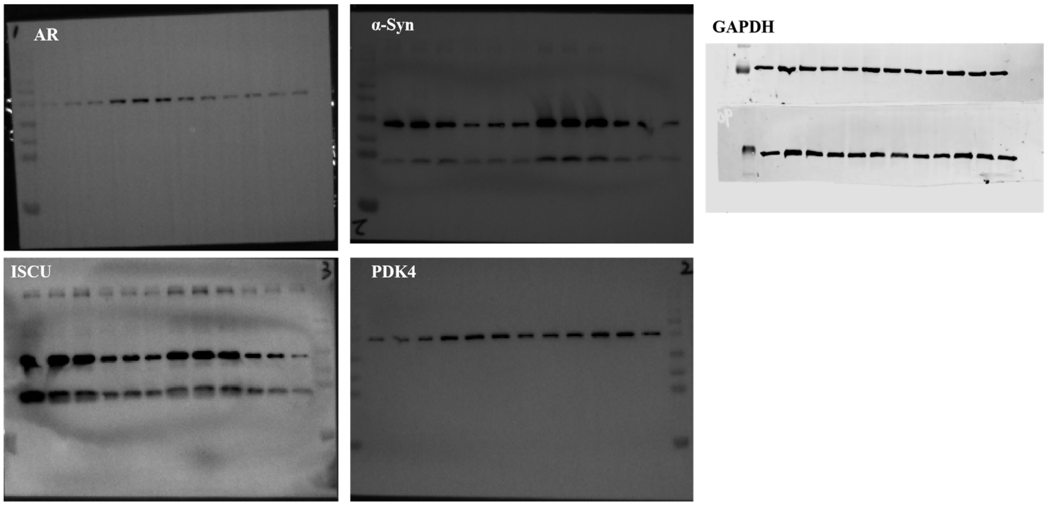

Figure S6. All raw blot and gel images.
